# Considerations for ultrasound exposure during transcranial MR acoustic radiation force imaging

**DOI:** 10.1038/s41598-019-52443-8

**Published:** 2019-11-07

**Authors:** M. Anthony Phipps, Sumeeth V. Jonathan, Pai-Feng Yang, Vandiver Chaplin, Li Min Chen, William A. Grissom, Charles F. Caskey

**Affiliations:** 1 Vanderbilt University Medical Center Department of Radiology and Radiological Sciences, Nashville, TN, USA; 2 Vanderbilt University Institute of Imaging Science, Nashville, TN, USA; 3 Vanderbilt University Department of Biomedical Engineering, Nashville, TN, USA

**Keywords:** Preclinical research, Biomedical engineering

## Abstract

The aim of this study was to improve the sensitivity of magnetic resonance-acoustic radiation force imaging (MR-ARFI) to minimize pressures required to localize focused ultrasound (FUS) beams, and to establish safe FUS localization parameters for ongoing ultrasound neuromodulation experiments in living non-human primates. We developed an optical tracking method to ensure that the MR-ARFI motion-encoding gradients (MEGs) were aligned with a single-element FUS transducer and that the imaged slice was prescribed at the optically tracked location of the acoustic focus. This method was validated in phantoms, which showed that MR-ARFI-derived displacement sensitivity is maximized when the MR-ARFI MEGs were maximally aligned with the FUS propagation direction. The method was then applied *in vivo* to acquire displacement images in two healthy macaque monkeys (*M fascicularis*) which showed the FUS beam within the brain. Temperature images were acquired using MR thermometry to provide an estimate of *in vivo* brain temperature changes during MR-ARFI, and pressure and thermal simulations of the acoustic pulses were performed using the k-Wave package which showed no significant heating at the focus of the FUS beam. The methods presented here will benefit the multitude of transcranial FUS applications as well as future human applications.

## Introduction

Researchers have known that ultrasound can affect neuronal activity for nearly a century^1^ and are increasingly exploring focused ultrasound (FUS) for neuromodulation, which is defined as reversible stimulation inhibition, modification and therapeutic alteration^2^. Transcranial thermal ablation with high intensity focused ultrasound is a mature technology with FDA approval for non-invasive ablative thalamotomy procedures in patients^3^. Pulsed-wave FUS at relatively low acoustic intensities is being developed as a research tool for non-invasive neuromodulation, with the ability to both stimulate and suppress neuronal activity through intact skull^4^. In addition to the direct effects of FUS neuromodulation, researchers are using FUS to locally deliver neuromodulatory drugs^5^, transiently open the blood brain barrier^6^, or deliver genetic vectors capable of modulating neural activity in spatially selective regions^7^.

Prior to applying FUS, knowledge of the location of the acoustic focus must be obtained to verify that the ultrasound beam is reaching its intended target. For ablative procedures, small temperature rises in the target tissue^8^ tracked with MR thermometry pulse sequences^9^ have been used to determine where the FUS beam is within the brain. However, off-target heating in the near- and far-fields of the ultrasound transducer can have deleterious effects^10,11^ and human brain function is known to be sensitive to fluctuations in brain temperature^12^. Given these concerns, a beam localization method that does not rely on temperature increases is desirable for FUS applications. Optical tracking has been used to target the FUS beam^13–15^. This method does not require any energy to be deposited in the brain but suffers from registration errors and does not account for aberrations induced by the skull. Simulations of the FUS transmitting through the skull can be performed with CT-derived acoustic property maps to estimate the focus location^16^. However, these parameter maps are both subject skull-specific^17^ and also depend on the X-ray energy and reconstruction kernel of the measured Hounsfield units (HU)^18^. Additionally, accurate simulations can require long computation times.

MR-acoustic radiation force imaging (MR-ARFI) pulse sequences can localize the acoustic focus prior to FUS procedures. In MR-ARFI, motion-encoding gradients are used to encode the tissue displacement response to a short ultrasound excitation (≈ms) into the phase of an MR image^19^. The acoustic radiation force is proportional to the local acoustic intensity of the ultrasound beam, so monitoring displacement via MR-ARFI provides a non-invasive tool to both localize the acoustic focus and to calibrate beam intensity. Unlike current beam localization methods, which predict the focus location based on simulations that are registered to the experiment, MR-ARFI is non-parametric and does not require *a priori* knowledge of skull acoustic properties, but rather can localize the beam *in situ* prior to any FUS application. MR-ARFI-derived displacement measurements have been validated in small animal *in vivo* studies, with ultrasound imaging-derived measurements as the gold standard^20^. Also, *ex vivo* studies in human cadavers have shown that sufficient sensitivity to displacement can be achieved beyond intact skull with MR-ARFI using commercial transcranial FUS transducers^21^.

In FUS applications like neuromodulation where the target tissue should not be affected by the beam localization procedure, MR-ARFI is constrained by the potential accumulation of ultrasound energy in the target tissue, which can have unwanted bioeffects. Broadly, interactions of ultrasound with tissue can be divided into either thermal or non-thermal mechanisms^22^. As ultrasound waves propagate through tissue, some energy is attenuated and absorbed in the form of heat, depending on factors like the local acoustic intensity, frequency, exposure time, and tissue type. This is especially relevant for transcranial applications of ultrasound on living subjects since their skulls are covered by skin, soft tissue and muscle. It is known that the attenuation coefficient of skull bone (up to 20 dB·cm^−1^·MHz^−1^) is many times that of brain tissue (approximately 0.6 dB·cm^−1^·MHz^−1^)^23^. Ultrasound also interacts with tissue via non-thermal mechanical interactions. Pressure waves with high rarefactional (negative) amplitudes can draw dissolved gas out of liquid tissue, forming cavitation bubbles. Cavitation bubbles may expand and contract with small-amplitude oscillations (stable cavitation), but in some cases, they may rapidly collapse and produce shock waves (inertial cavitation), which can lead to tissue damage. While safety limits have been proposed in the context of diagnostic ultrasound imaging applications, they likely do not encompass the range of biological phenomena that may occur in response to pulses commonly used during MR-ARFI. To safely implement MR-ARFI, thermal and mechanical bioeffects must be avoided while applying high enough ultrasound intensity to generate detectable displacements. The goal of the present study is to detect and visualize the transcranial FUS beam with MR-ARFI and ultimately establish a framework to understand potential bioeffects based on simulations and experimental observations.

We implemented transcranial MR-ARFI in living non-human primates informed by simulations of pressure fields and thermal deposition in the skull and brain. To maximize displacement sensitivity, we developed an optical tracking method to ensure that the MR-ARFI motion-encoding gradients are aligned with the FUS propagation direction and with the imaged slice prescribed at the optically tracked location of the acoustic focus. The methods described here address the need to determine the precise location of the FUS’s interaction with brain tissue during transcranial FUS stimulation. We present these methods in the context of minimizing FUS exposure during FUS neuromodulation, where freely moveable transducers are increasingly used creating a need to image the estimated focal location with MR-ARFI and determine the gradient direction that will minimize exposure. The techniques and bioeffects considerations presented here are generally applicable to all transcranial FUS procedures.

## Results

### Skull attenuation and pressure field simulations

**Figure 1:**
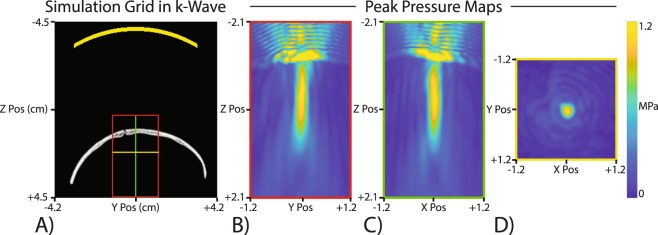
Pressure simulation with an *ex vivo* skull fragment. (**A**) The full simulation grid with the transducer location represented in yellow at the top and the skull fragment CT which was used to generate the medium properties. The inset boxes are where thermal simulations were performed. (**B**–**D**) Peak pressure maps for three intersecting planes at the focus location. The highest pressure was within the skull itself, but the pressure maps are saturated to better show the focus. The maximum pressure at the focus target was 1.14 MPa and the maximum pressure within the skull was 3.19 MPa.

**Figure 2:**
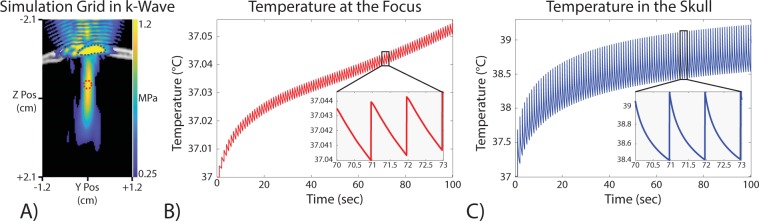
Thermal simulation results of 100 MR-ARFI sonications. (**A**) The thermal simulation grid with a pressure map overlay. The pressure map is thresholded to only show values above 0.5 MPa. The red circle shows the approximate target location and the blue outline shows the maximal heating area reported in the skull. (**B**) The temperature at the focus target increased by less than 0.1 °C. The target was located at the intersection of the three slices in (**A**). (**C**) The voxel with the largest temperature change in the simulation. This voxel is located within the skull as expected due to the much higher acoustic absorption and pressure. This simulation showed a temperature change within the skull of up to 2.2 °C.

Measurements of the FUS transducer output were acquired with and without an *ex vivo* skull present to characterize attenuation expected in the skull. The mean detected pressure at 802 kHz in the water tank were 284 kPa (free-field) and 91 kPa (skull fragment present). With the skull fragment, 32.1% of the free-field pressure was recorded at the same location beyond the skull. Simulations of the same FUS pulses used during subsequent MR-ARFI showed a maximum pressure at the target of 1.14 MPa and a maximum pressure in the skull of 3.19 MPa (Fig. 1, note the color map is scaled to emphasize the focus in the brain). Since the highest pressure is in the skull, which absorbs more sound than tissue, thermal simulations show the most heating in the skull (Fig. 2). A 100-sonication simulation of an ARFI pulse resulted in a maximum heating in the skull of 2.22 °C and a maximum heating at the target of 0.05 °C. The heating in the skull approached a steady state; however, at the focus, the heating did not appear to be in a steady state. An additional pressure simulation matched to the conditions of the water tank experiment resulted in a free field simulated pressure of 284 kPa and transcrainial pressure of 118.5 kPa. This simulation showed 41.73% transmission through the *ex vivo* skull fragment.

### Aligning MR-ARFI MEGs with FUS propagation direction improves sensitivity

**Figure 3:**
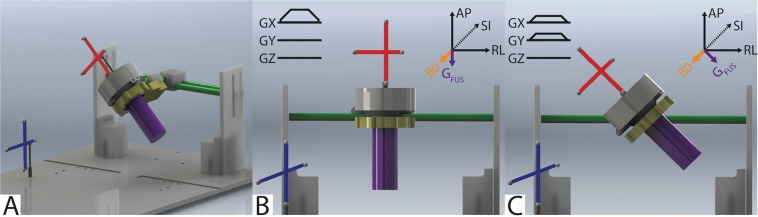
Targeting with an optically tracked FUS transducer. (**A**) A spherically-focused single-element FUS transducer (gray) was used to sonicate a tissue-mimicking brain phantom (purple). An MRI-compatible rigid body tracker was mounted to the patient bed (blue), and another body tracker was mounted to the transducer (red). As shown, the phantom mold was rigidly attached to the transducer housing. The transducer-phantom apparatus was mounted on a three-axis stereotactic frame (green) so that sonications could be performed in any physical orientation. (**B**,**C**) Demonstrate how the location of the transducer was obtained via optical tracking and used to align the MR-ARFI motion-encoding gradients (MEGs) with the FUS propagation direction (G_FUS_).

**Figure 4:**
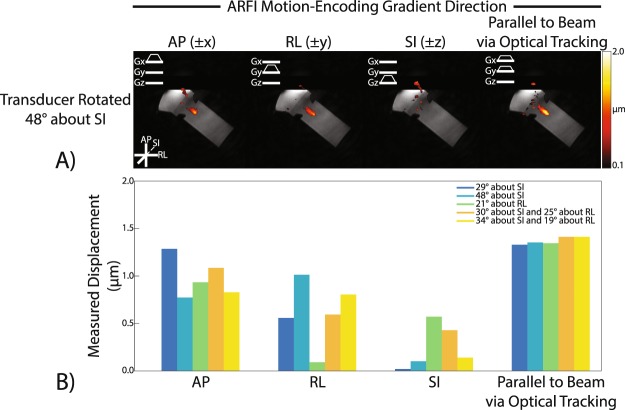
Optical tracking-based alignment of MR-ARFI motion-encoding gradients (MEGs) with the FUS propagation direction in a phantom. (**A**) MR-ARFI displacement maps for an oblique rotation of the FUS transducer. Maps were shown with the MEGs prescribed along the cardinal axes and along the optical tracking-determined propagation direction. (**B**) Mean displacement measured by MR-ARFI at the focus for each MEG orientation and each transducer rotation. The highest mean displacement is detected when the MEG is aligned with the FUS propagation direction obtained via optical tracking. Mean focal displacement was computed in a 3 × 3 px ROI at the acoustic focus.

To test whether optical tracking could predict MEG angle, we placed the transducer at varied angles relative to MEG direction and applied sound to a phantom known to absorb sound and deform similarly to tissue (Fig. 3). Displacement maps acquired in the agar and graphite phantom using our optically tracked MR-ARFI pulse sequence in each MEG and transducer/phantom orientation show micron-scale displacement at the expected location of the ultrasound focus (Fig. 4a). Figure 4B reports mean focal displacements in a 3 × 3 pixel ROI at the focus for each MEG and transducer orientation pair. In every transducer orientation, the measured displacement was highest when the MEGs were prescribed along the FUS propagation direction using the optical tracking method. At matched acoustic output, the range of detected displacements was low when MEGs were prescribed via optical tracking (1.33–1.41 μm; mean ± SD = 1.37 + 0.04 μm), suggesting that the optically-tracked alignment of the MEG can be used to improve SNR.

### MR-ARFI in non-human primate brain

**Figure 5:**
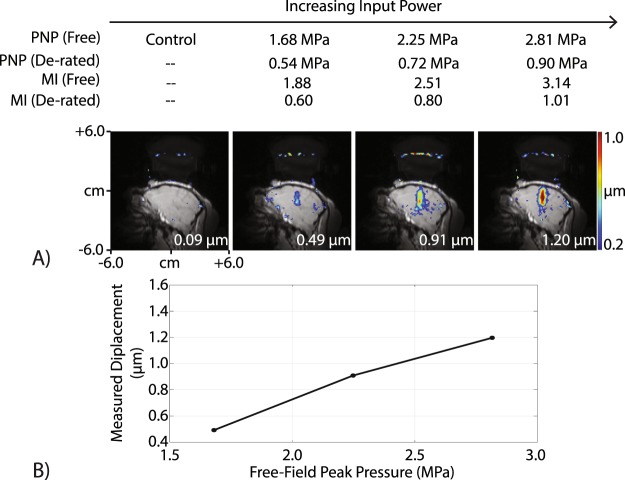
Transcranial displacement images in a living macaque. (**A**) Displacement images were obtained with our optically tracked MR-ARFI pulse sequence *in vivo*, and the peak transcranial displacement is plotted in (**B**). The measured displacement increased with increasing pressure. At the lowest pressure tested (estimated 0.54 MPa in the brain), a 0.49 µm displacement was obtained. This demonstrates that detectable displacement is feasible at pressures that are not expected to generate cavitation in the brain.

**Figure 6:**
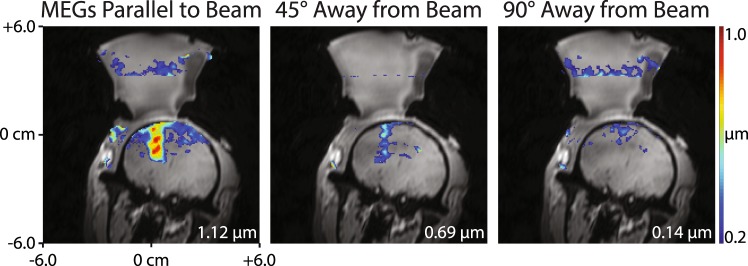
Optical tracking-based alignment of MR-ARFI MEGs with the FUS propagation direction *in vivo*. Displacement images were acquired with the MEGs aligned parallel to the beam (left), 45° away from the beam (middle), and 90° away from the beam (right). When the MEGs were prescribed off-axis, the measured displacement reduced, indicating that MR-ARFI in living subjects requires proper MEG alignment to achieve optimal sensitivity to displacement.

Figure 5 shows transcranial displacement images acquired using our optically tracked MR-ARFI pulse sequence in a living macaque. We measured a mean focal displacement of 1.20 µm at the highest acoustic power that was applied, which corresponds to a de-rated peak negative pressure (PNP) of 0.90 MPa in the brain (free-field PNP = 2.81 MPa). Using the MEG orientation determined by optical tracking, we reduced the power and measured decreasing displacement values to estimate the detection threshold during *in vivo* imaging with MR-ARFI. The smallest displacement we detected was 0.49 µm at a power level generating a de-rated PNP of 0.54 MPa in the brain (free-field PNP = 1.68 MPa). We acquired additional displacement images in one living macaque with the MEGs oriented away from the FUS propagation axis and a de-rated PNP of 0.72 MPa (free-field PNP = 2.25 MPa), which are shown in Fig. 6. With the gradients rotated off-axis, the mean focal displacement decreased from 1.12 µm (parallel to the beam) to 0.69 µm (45° away from the beam) and 0.14 µm (90° away from the beam).

**Figure 7:**
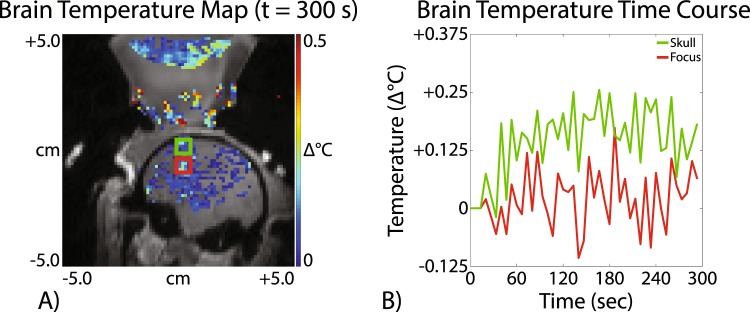
MR thermometry in a living macaque using MR-ARFI-based acoustic parameters. (**A**) A representative *in vivo* brain temperature map shows that no significant temperature rise could be detected using acoustic parameters designed for MR-ARFI. (**B**) The brain temperature time course at the acoustic focus (red) and near the skull (green) is shown. Mean focal temperature was computed in a 3 × 3 px ROI.

A representative brain temperature image acquired in one living macaque at a de-rated PNP of 0.72 MPa (free-field PNP = 2.25 MPa) is shown in Fig. 7A. A plot of the mean focal temperature is shown in in red in Fig. 7B and the mean temperature near the skull is in green. These results indicate that no significant brain temperature rise could be detected at the focus via MRI-based temperature monitoring when acoustic parameters designed for MR-ARFI were used. In the brain near the skull we detected approximately a 0.2 C rise. We did not observe macroscopic evidence of cavitation-induced skin lesions in either monkey in the region where the FUS entered the skull.

## Discussion

### MR-ARFI in the non-human primate brain

Through simulation and phantom studies, we identified FUS parameters that can be used to transcranially induce displacements in brain tissue and developed methods to measure this displacement with MR-ARFI. We used this system to non-invasively localize the focus of a therapeutic FUS transducer by measuring ARF-induced displacements within a 4-minute scan time in living macaque brains at 7 T MRI. Our work demonstrates the feasibility of using MR-ARFI to map the FUS beam transcranially in a large animal and localize its focus in conjunction with structural imaging-based neuronavigation via optical tracking. Much prior work has established MR-ARFI in phantoms^19^; our study demonstrates transcranial MR-ARFI in a survival imaging session in the brain of a large animal with intact skull with surrounding tissues of skin, soft tissue, and muscle. In implementing this imaging protocol, we also highlight important design aspects that must be considered when developing transcranial MR-ARFI protocols in large living animals and eventually humans.

### Measuring *in vivo* displacement using MR-ARFI with negligible heating

During the MR-ARFI sequence, a low duty cycle (e.g. long TR and short FUS pulse) was required to avoid heating that could lead to adverse bioeffects in the brain, the skull, and the scalp. In our study, we minimized heating by using the lowest FUS intensity and shortest pulse duration needed to generate detectable displacement and separating the FUS pulses in time with a TR of 1 second (overall duty cycle of 0.23%). The TR must be short enough to acquire a displacement map in a practical time frame. While tissue damage can occur with large temperature changes it is possible that even small temperature changes in the brain can change neurological function temporally, which would be undesirable during neuromodulation studies^12^. In our study, heating in the brain was less than ~0.1 °C in MR thermometry images derived from phase maps acquired during MR-ARFI, which is consistent with simulations. In the skull, our simulations predicted that a ~2 °C temperature rise may be possible. With proper consideration of duty cycle, our study shows displacement maps in the brain can be generated in a feasible scan time (<5 minutes) with <0.1 °C heating.

To minimize thermal bioeffects, the Food and Drug Administration (FDA) mandates safety limits on the acoustic output levels of clinical ultrasound transducers^24^. The spatial peak-temporally averaged intensity (I_SPTA_), which gauges the likelihood of heating, should not exceed 720 mW·cm^−2^. Although these metrics are designed around imaging transducers and pulses, they provide a basic guideline. The pulsing scheme used in our implementation of MR-ARFI is lower than these limits for diagnostic imaging. Combined with our simulations and the absence of detectable heating with MR thermometry, we conclude that heating from ARFI pulses can be reduced to a negligible amount while generating high quality images.

### Simulated peak negative pressure within and near the skull

Our study suggests that high peak negative pressures that form at the surface and potentially within the skull present the highest risk of potential bioeffects during MR-ARFI. Cavitation is the process of bubbles forming from small gaseous nuclei and subsequently collapsing, generating forces capable of severely damaging tissue^25^. Our retrospective simulation of the an FUS pulse with intensity required to produce an approximate 1 μm displacement through the skull show pressures as high as 3.2 MPa (MI ~ 3.5) within or at the surface of the skull, suggesting that cavitation within or near the skull is a realistic concern. We note that our simulations do not encapsulate all possible acoustic interactions, and specifically may be inaccurate within the skull where our CT voxel size is too large to represent microstructure. A prior study of FUS interactions with the microstructure of the skull show that wavelength size heterogeneities can cause internal scattering and shear absorption that may ultimately reduce the PNP within the skull and reflect less uniformly than our simulations suggest^26^. Additionally, simulations have been shown to underestimate the pressure beyond the skull if the simulation grid spacing is not at least 20 times the wavelength due to a staircasing effect of the skull geometry^27^. Our hydrophone measurements acquired in a water bath compare favorably with simulations, but error associated with staircasing or inability to represent microstructure remain challenging. While no evidence of cavitation-induced skin damage was observed in either monkey on a macroscopic scale, tissue samples will be collected for further pathological analysis at the conclusion of our neuromodulation experiments.

Cavitation is a stochastic process that is difficult to predict, since it depends on many conditions (presence of cavitation nuclei, frequency and pressure)^28^. The probability of cavitation is proportional to the peak negative pressure and inversely proportional to the frequency^29^. During diagnostic ultrasound imaging, the MI is used to gauge the likelihood of cavitation activity and is defined as the peak negative pressure (PNP) in MPa divided by the square root of the center frequency in MHz; it should not exceed 1.9 during shot pulses used during diagnostic ultrasound^24^. Most safety studies for ultrasound have been performed for imaging pulses which generally have higher frequency and use shorter pulses compared to MR-ARFI pulses. Cavitation-induced damage could be more likely when using millisecond-long pulses as required for ARFI, since these pulses provide repeated negative pressure cycles that would potentially drive bubble oscillation and generate repeated cavitation events. The maximum MI of all de-rated FUS pulses in the brain used in our study (estimated from hydrophone measurements with re-hydrated *ex vivo* skull) was 1.0. The free-field pressures exceed limits on MI for imaging, which, combined with known subject-to-subject variations in skull attenuation, approaches regimes where cavitation is possible.

### Magnitude of the ARF-induced displacement

We detected displacements with magnitude less than 1 μm, which is consistent with other reports of MR-ARFI in living animals^30–32^. It is helpful to consider our detected displacement in the context of direct observations of displacement due to radiation force. A majority of analysis of the magnitude of ARF-induced displacement has been done at frequencies greater than 2 MHz to understand displacements during ultrasound imaging-based ARFI; however, rough comparisons can be made to the 802 kHz pulses in our study since the radiation force is proportional to absorption and scales with frequency:

$${F}_{rad}\propto \frac{\alpha {I}_{sppa}}{c},$$

where α is the absorption of the medium, I is the spatial peak average intensity of the ultrasound pulse, and c is the speed of sound in the medium^33^. One study has used high-speed microscopy to directly observe displacement of a microbead due to acoustic radiation force in a tissue-like phantom, measuring a displacement of 80 μm in response to a 5 MHz pulse (reported I_sppa_ = 2500 W/cm^2^, and MI = 1.8)^34^. In our study, the I_sppa_ was approximately 185 W/cm^2^ with a center frequency of 802 kHz. Since the intensity and frequency (and hence attenuation) in our study are both approximately 10X lower than the direct observation study, we expect radiation forces in our study to be two orders of magnitude lower. Assuming that micron-scale displacements follow Hooke’s law so that force is proportional to displacement, our observed displacement of 1 μm is expected.

Error in the optically tracked location and angle of the FUS beam can lead to reduced measured displacement and error in slice selection. In previous work^35^, we estimated that optical tracking could localize the acoustic focus with an accuracy of approximately 3 mm with an estimated error of 1.4 degrees in angulation. For small errors in angle, the measured displacement will have nearly full amplitude. Because ARF-induced displacement decreases with increasing distance from the focus, slices must be selected that encompass the beam. In our study, the angle and location provided by optical tracking guided slice selection, which reduces overall FUS exposure since each MR-ARFI slice requires multiple FUS bursts.

### Imaging time, gradient strength, and minimizing FUS exposure

Localizing the ultrasound focus with MR-ARFI should ideally deposit as little FUS energy as possible. Maximizing sensitivity of the MR sequence allows for detection of smaller displacements for a fixed acoustic intensity. To encode micron-scale displacements, MEGs with high gradient strengths and long durations are required to accrue detectable phase shifts into the reconstructed MR-ARFI displacement image. To maximize sensitivity to the ARF-induced phase change, MR-ARFI is typically implemented by complex phase subtraction of two spin echo MR acquisitions obtained with switched polarity MEGs^19^. Additional subtraction of an acquisition without ultrasound application has been shown to minimize motion-induced phase contributions unrelated to the ARF (e.g., respiration)^36^. Echo-planar imaging (EPI)-based sequences have also been used to rapidly encode displacement images while minimizing ultrasound energy deposition^37^. In this work, we further developed the MR-ARFI pulse sequence using an optical tracking system to predict the transducer orientation so that the MR-ARFI MEGs could be prescribed along the FUS propagation axis and the slice could be located at the predicted focus. We showed in tissue-mimicking brain phantoms (Fig. 4) and living macaques (Fig. 6) that knowledge of the transducer orientation can improve displacement sensitivity without requiring any additional sonications. In our experience, a spin echo multi-shot EPI acquisition strategy provided the best balance between scan time and image quality for our application. Previous efforts in MR-ARFI pulse sequence development might be considered for future directions. Both single-shot EPI^37^ and steady‐state free precession^38^ pulse sequences have been proposed to further reduce scan time for MR-ARFI, though these acquisitions are highly sensitive to geometric distortions, especially at 7 T. Encoding schemes that use bipolar gradients^36^ or alternating triggers to the transducer (i.e., triggering the sonication on either the forward gradient or the gradient rewinder)^39^ have also been shown to improve phase stability. Volumetric imaging strategies for MR-ARFI have also been proposed^40–42^. A custom surface coil was used for transmit/receive due to the lack of an integrated body/volume coil in our 7.0 Tesla MRI scanner. We fabricated a 6 cm surface coil integrated with the transducer’s coupling cone specifically for this imaging application so that the SNR would be maximized near the acoustic focus in the desired target location of our non-human primate subjects. In our *in vivo* MR-ARFI acquisitions, we obtained an SNR of 14.94 (where SNR = peak focal displacement/STD of noise displacement), which was sufficient to clearly observe the acoustic focus *in vivo*.

### Propagation direction, frequency, and f-number

Moderately focused spherical cap transducers are typically used during FUS neuromodulation studies, opposed to hemispherical arrays used during ablation. A moderately focused transducer, as used in our study, generates an acoustic radiation force primarily in the propagation direction, and similar approaches may not be feasible with hemispherical arrays which would generate increased PNP that does not contribute to the ARF in the direction perpendicular to MEGs but would contribute to both thermal and cavitation-related bioeffects. However, non-linearities (e.g. increased peak pressures) build at the focus more easily with moderately focused transducers compared to hemispheres^43^. Center frequency is another important consideration. Increased center frequencies generate greater force due to increased absorption, while neuromodulation literature suggests that lower frequencies elicit neural responses at lower pressures^44^. A center frequency capable of both MR-ARFI and neuromodulation would be ideal, but a trade-off is required between the abilities to focus through the skull, elicit neuromodulation and displace tissue by a detectable amount. In this study, we used a transducer with multiple resonance peaks so that ARFI was performed at a high frequency while neuromodulation could be performed at a lower frequency without changing the transducer position^45^. To precisely map the neuromodulation beam, further considerations would need to be made about skull interactions at the different frequencies.

## Conclusion

This work has demonstrated that MR-ARFI can be used to map and localize a neuromodulation ultrasound beam in the living brain with no detectable negative bioeffects in 5 minutes. Our associated simulations and analysis highlight parameters that should be considered in designing MR-ARFI for minimal FUS exposure. Further optimization of the MR sequence will allow for lower ultrasound intensities and durations to be used which will lower risk of damage and remove confounding factors for research studies. This work describes a method to visualize the acoustic beam in the skull, which will directly benefit the field of FUS neuromodulation and drug delivery.

## Materials and Methods

### Characterizing transcranial focused ultrasound

A spherically-focused, single-element piezoceramic transducer was used for all experiments (H115MR, Sonic Concepts, Bothell, WA). It has a geometric focus of 63.2 mm and opening diameter of 64 mm. Sonications were performed at its third harmonic of 802 kHz. A custom 3D printed coupling cone with a 3 cm aperture held the transducer. Acoustic data were collected with a needle hydrophone (HNC 0400, Onda Corp., Sunnyvale CA). The free-field pressure from the FUS transducer was measured for a series of input voltages up to a mechanical index (MI) of 1.2. A calibration curve was determined from this data set which was used to estimate pressures used for MR-ARFI.

To estimate the attenuation induced by the NHP skull, we measured the pressure field behind a rehydrated *ex vivo* macaque skull piece. The skull piece is from the top of the head and is approximately 7 × 6 × 3 cm in dimension with a thickness between 2 and 3 mm. The skull piece was placed in degassed water for 24 hours prior to the measurements. The FUS transducer was coupled to a water tank through an acoustic window, and the needle hydrophone’s voltage was recorded with the transducer driven at 802 kHz. The pressure during transcranial sonications was then measured at the free-field focal location for 5 different positions of the skull to account for variations in thickness and incident angle. The transmission percentage was taken to be the ratio of pressure measured with and without the skull present. This transmission percentage was used to derate our free field pressure values to estimate the focal pressure within the skull.

### Acoustic and thermal simulations of ARFI pulses

A numerical model of a NHP skull surrounded by brain tissue was built using the k-Wave package^46^ for pressure and thermal simulations. A CT scan of the *ex vivo* NHP skull fragment was acquired on a clinical PET/CT scanner (Philips Vereos PET/CT, Philips Healthcare, Best, NL). The skull was placed in degassed water for 48 hours and then embedded in 1% agar. The scans were collected at 140kVp and 300 mAs and had a resolution of 0.19 × 0.19 × 0.67 mm^3^. The images were reconstructed with soft tissue (filter type ‘B’) and bone (filter type ‘YC’) filters. The image volume was resampled using the imresize3 function in MATLAB (Mathworks, Natick, MA, USA) to isotropic 0.3 mm voxels. Both the soft tissue and bone CT reconstructions were in Hounsfield units (HU). A histogram with 400 bins of size approximately 7 HU (which varied depending on initial HU minimum and maximum values) was generated. A maximum HU value to be used was determined from the histogram data based on the HU value of the highest bin with at least 500 voxels. This method was used so that the resulting density and speed of sound maps had values similar to previously reported values. The CT data was then compressed so that all values below 0 HU were mapped to 0 HU and all values above the maximum threshold were remapped to that value (1632 HU for the bone reconstruction filter). The soft tissue filter was used to generate a mask of the skull fragment to apply acoustic absorption, thermal conductivity, and specific heat in the simulations (Table 1). Other acoustic properties were generated from the CT images reconstructed with the bone filter using a method similar to Aubry *et al*.^16^. The porosity was estimated for each voxel as ϕ_i_ = 1 − (HU_i_/max(HU_volume_)). This value was then used to calculate a speed of sound and density for each voxel ρ_i_ = ϕ*ρ_water_ + (1 − ϕ)*ρ_bone_; c_i_ = (c_Max_ − c_Min_)*(1 − ϕ) + c_Min_ where ρ_water_ = 1000 kg·m^−3^, ρ_bone_ = 2100 kg·m^−3^, c_min_ = 1500 m·s^−1^, and c_max_ = 2900 m·s^−1^. All parameter maps were padded to a grid size of [Nx,Ny,Nz] = [300,280,280].

**Table 1:** Thermal parameters and acoustic absorption values for the skull and the rest of the simulation grid.

Parameter	Skull	Grid
Absorption (dB/cm/MHz)	8	0.4
Thermal Conductivity (W/m/K)	0.3	0.5
Specific Heat (J/kg/K)	1700	3600

The H115MR transducer was modeled in k-Wave and placed so that the geometric focus was approximately 1 cm past the inner surface of the skull fragment. Thermal simulations presented in this paper represent a retrospective simulation of our *in vivo* experiments. An 80-cycle pulse was used as the input source for the transducer. The amplitude was matched so that a free-field simulation was the same as the highest estimated free-field pressure used *in vivo* (methods described below). An additional pressure simulation was performed to compare our simulation results with our water tank measurements behind the skull fragment. For this simulation the free field pressure was matched to the measured free field pressure in the water tank (284 kPa) and then a simulation was performed through the skull to simulate the transmission loss in a water tank. A GPU-accelerated 3D k-Wave simulation was run on a workstation PC (HP Z820, Xeon E5, with 256 GB RAM, Hewlett Packard, Palo Alto, CA) with a 16 GB Nvidia Titan GPU (Nvidia, Santa Clara, CA). The maximum pressure was recorded for every voxel in the simulation grid.

Heating predictions were made by solving the Pennes’ bioheat equation as implemented in the k-Wave package. The maximum pressure and acoustic absorption from the pressure simulation were used to scale the volume rate of heat deposition for the bioheat simulation. This simulation implemented the bioheat equations in a region around the focus that contained the part of the skull fragment that most of the sound passed through (a grid size of [Nx,Ny,Nz] = [140,80,80] with 0.3 mm spacing in all dimensions). The simulated ARFI pulse duration and duty cycle was matched to that used during MR-ARFI (4.5 ms pulses with a 1 Hz pulse repetition frequency (PRF)). A time step of 0.1 ms was used during the 4.5 ms ARFI pulses, while a longer time step of 5 ms was used during the “off” periods to ensure feasible computation time. 100 sonications were simulated and the temperature was recorded at each point in the grid for each time point.

### MR-ARFI pulse sequence

All MR imaging was performed on a 7.0 T Philips Achieva human research scanner (Philips Healthcare, Best, NL). Displacement images were acquired using an optically tracked 2D spin echo MR-ARFI pulse sequence. Unipolar trapezoidal motion-encoding gradients (MEGs) were placed before and after the refocusing RF pulse to generate ARFI contrast^19^. The MEGs were set to 3 ms in duration with the scanner’s maximum gradient strength (40 mT·m^−1^) on their plateaus, which resulted in low diffusion-weighting (b-value ≈ 9.3 s·mm^−1^). Imaging parameters were: 12.0 × 12.0 cm^2^ FOV; 60 × 60 matrix; 2.0 × 2.0 mm^2^ voxel size; 1 slice; 2.0 mm slice thickness; echo time (TE)/repetition time (TR) 17/1000 ms; 2D multi-shot echo-planar imaging (EPI) readout with 5 lines per TR. A custom 6 cm surface coil integrated with the transducer’s coupling cone was used for transmit/receive. In each TR, a TTL pulse was sent from the scanner to the FUS waveform generator to trigger a sonication. Sonications were synchronized with the rewinder MEG using a trigger offset of −2 ms to allow displacement to reach a steady state^32^. Sonications were performed at 802 kHz for 4.5 ms (3609 cycles) with an acoustic pressure (maximum free field of 2.81 MPa) that would not be expected to exceed a temperature increase greater than 1 °C or MI greater than 1.1 within the brain based on the previously described acoustic simulations and hydrophone experiments. Four phase images with switched polarity MEGs and with or without a sonication were acquired in an interleaved fashion (*ϕ*^*ON*+^, *ϕ*^*OFF*+^, *ϕ*^*ON*−^, *ϕ*^*OFF*−^). Each phase image was acquired with five averages. Since the ultrasound PRF is specified by the TR of the pulse sequence, we used a relatively long TR of 1000 ms (1 Hz PRF) to maintain a low duty cycle. In total, 120 sonications were performed at a duty cycle of 0.23%, with a total scan time of 4.0 minutes to produce one displacement image. Displacement images were reconstructed using complex phase subtraction ($\Delta x={\rm{\angle }}({\varphi }^{ON+}\cdot {({\varphi }^{ON-})}^{\ast }\cdot {({\varphi }^{OFF+}\cdot {({\varphi }^{OFF-})}^{\ast })}^{\ast })/2\gamma G\tau$, where *γ* is the gyromagnetic ratio, *G* is the gradient strength, and *τ* is the gradient duration). Images were reconstructed offline in MATLAB 2017a (MathWorks, Natick, MA).

Since the transducer is freely-movable and manually positioned over the targeted region, prescribing the MEGs for MR-ARFI requires precise knowledge of the slice offset and angulation of the transducer along the anterior-posterior (AP or ±x), right-left (RL or ±y), and superior-inferior (SI or ±z) cardinal axes. We used optical tracking to ensure that the MEGs were aligned with the FUS propagation direction and that the imaged slice was prescribed at the optically tracked location of the acoustic focus. Previous efforts have described how optical tracking can be used to estimate the focus location and target the FUS beam^13,35^. This procedure uses a Polaris Vicra optical tracking system (Northern Digital Inc., Ontario, CAN) and is illustrated in Fig. 3. An MRI-compatible rigid body tracker is mounted to the patient bed and serves as the global reference location. Another body tracker is mounted to the transducer as the tracked location. Multimodality fiducial markers (IZI Medical Products, Maryland, USA) are placed near the focus location. The fiducials are localized in image space using a 3D fast spoiled gradient-recalled echo T1-weighted high-resolution isotropic volume examination (THRIVE) pulse sequence (voxel size 0.4 × 0.4 × 1 mm^3^, TE/TR 1.89 ms/4 ms). The fiducials are manually identified in the T1-weighted image stack using 3DSlicer (http://www.slicer.org/). In front of the optical tracking camera, the fiducials are localized in physical space using a reflective positioning stylus and recorded in 3DSlicer. Finally, these are registered to the fiducials’ image locations, yielding a physical-to-image space transform. The transducer can then be freely rotated in physical space, with 3DSlicer reporting the slice offset and angulation required to prescribe the MR-ARFI scan with maximum displacement sensitivity.

### Optically tracked MR-ARFI in phantoms with simulated targeting

To simulate the targeting of arbitrary brain regions with our optically tracked MR-ARFI pulse sequence, and to demonstrate the need to align the MR-ARFI MEGs with the FUS propagation direction via optical tracking, displacement images were acquired in an *ex vivo* agarose phantom designed to mimic brain tissue acoustic properties (1% agarose, 4% graphite, and 10% n-propanol in water)^47^. Gel based phantoms typically have a lower limit for stiffness around 10kPa^48^ while brain tissue is much lower near 1 kPa^49^. Based on this difference, we do not expect the relative magnitudes of ARF-induced displacement in brain tissue and this phantom to be comparable, and thus do not compare the magnitude of displacement of the phantoms to tissue. For these experiments, the transducer housing was rigidly attached to a cylindrical phantom mold, and the transducer-phantom apparatus was mounted on a plastic tabletop with a three-axis stereotactic frame. In this way, our sonications could be targeted in any physical orientation. Targeting of the transducer-phantom apparatus is demonstrated in Fig 3B,C. To fabricate the phantom, 5 grams of food-grade agarose powder was added to a 450 mL beaker of cold water. The beaker was heated in a microwave until it boiled. Twenty grams of 400 grit graphite powder (Panadyne Inc, Montgomeryville, PA) was then added, and after about 5 minutes of cooling, 50 mL of n-propanol was added to the agarose-graphite phantom mixture. The transducer housing was partially filled with 1% agarose in water and allowed to set before the phantom mixture was poured into the housing and phantom mold.

Displacement images were acquired using our optically tracked MR-ARFI pulse sequence after translating and rotating the transducer to a slice offset and angulation about the AP, RL, and/or SI cardinal axes. The transducer was positioned in one of six physical orientations: No rotation; 29° about SI only; 48° about SI only; 21° about RL only; 30° about SI and 25° about RL; and 34° about SI and 19° about RL. Four displacement images were acquired per transducer orientation: MEGs aligned along AP only; RL only; SI only; and aligned with the FUS propagation axis as determined by optical tracking.

### Optically tracked MR-ARFI in living non-human primates

Two healthy adult female macaque monkeys (*M fascicularis*) were scanned with the approval of the Institutional Animal Care and Use Committee (IACUC) at Vanderbilt University and in accordance with all relevant guidelines and regulations. For these experiments, a previously developed experimental platform for targeted ultrasonic neuromodulation in non-human primates was used^13,35^. Animals were sedated and positioned in a three-axis stereotactic frame with consistent physiological monitoring for the duration of the experiments. The location of the FUS beam was first determined using the same optical tracking workflow performed for our phantom experiments. This information was used to target the transducer on the right somatosensory network (S1 areas 3a/3b). Transcranial displacement images were acquired with our optically tracked MR-ARFI pulse sequence, ensuring that the MEGs were aligned with the FUS propagation direction and with the imaged slice prescribed at the optically tracked location of the acoustic focus. In one living macaque, we acquired additional displacement images aligned with the beam but with the acoustic pressure reduced by 20% and 40%, to provide an estimate of displacement sensitivity at low acoustic powers. As a negative control, we also acquired displacement images in one living macaque with the MEGs oriented off axis (i.e., 45° and 90° away from the FUS propagation direction).

In a separate experiment, sonications were performed in one animal using the same acoustic parameters for MR-ARFI, but monitored with an MR thermometry pulse sequence to provide an *in vivo* estimate on brain temperature changes during MR-ARFI. Temperature images were acquired using a 2D gradient-recalled echo thermometry pulse sequence^9^. Imaging parameters were: 10.0 × 10.0 cm^2^ FOV; 50 × 50 matrix; 2.0 × 2.0 mm^2^ voxel size; 5 slices; 2.0 mm slice thickness; TE/TR 10/25 ms; 2D single-shot EPI readout. Temperature images were reconstructed in MATLAB using the hybrid multibaseline subtraction and referenceless method^50^.
